# Draft Genome Sequence of a Kazachstania humilis Strain Isolated from Agave Fermentation

**DOI:** 10.1128/mra.01154-21

**Published:** 2022-03-02

**Authors:** Luis F. García-Ortega, Maritrini Colón-González, Iván Sedeño, Erick Santiago-Garduño, J. Abraham Avelar-Rivas, Manuel R. Kirchmayr, Alexander DeLuna, Luis Delaye, Lucia Morales, Eugenio Mancera

**Affiliations:** a Departamento de Ingeniería Genética, Centro de Investigación y de Estudios Avanzados del IPN, Unidad Irapuato, Irapuato, Mexico; b Laboratorio Internacional de Investigación sobre el Genoma Humano, Universidad Nacional Autónoma de México, Querétaro, Mexico; c Unidad de Genómica Avanzada (Langebio), Centro de Investigación y de Estudios Avanzados del IPN, Irapuato, Mexico; d Área de Biotecnología Industrial, Centro de Investigación y Asistencia en Tecnología y Diseño del Estado de Jalisco A.C. (CIATEJ), Zapopan, Jalisco, Mexico; University of California, Riverside

## Abstract

The ascomycetous yeast Kazachstania humilis is an active species in backslopped sourdough and in the spontaneous fermentation of several traditional foods and beverages. Here, we report the draft genome sequence of a *K. humilis* strain isolated from agave must from a traditional distillery in Mexico.

## ANNOUNCEMENT

The ascomycetous yeast Kazachstania humilis (Candida humilis, Candida milleri) is an abundant fungus during the preparation of a variety of fermented foods and beverages (1–5). Most reported isolates are from Asia and Africa, but there are also occurrences in North America (Global Biodiversity Information Facility database), including strains from a natural tequila fermentation in Mexico (6). Despite its wide geographical distribution and its potential for food and beverage production, there is little information about the genome of this species. Here, we report the complete genome assembly of a strain of *K. humilis* that was isolated from fermenting agave must in a traditional distillery in Mexico. The alcoholic beverages produced in these distilleries, such as mezcal, are obtained from the distillation of cooked and fermented juice of *Agave* sp. plants. These artisanal processes are characterized by open and “spontaneous” fermentations in which producers use no commercial inoculum, thus relying on environmental microorganisms.

The *K. humilis* strain (YMX004033) was isolated from a fermentation of cooked agave juice from a distillery located in the Mexican state of Zacatecas. For strain isolation from nitrogen-frozen must containing 25% glycerol, 1 mL was used to inoculate 20 mL of *Saccharomyces* *sensu stricto* enrichment medium containing 6% ethanol (7). When signs of microbial growth were detected at 25°C, a 10^−5^ dilution was plated in Wallerstein Laboratory (WL) medium (Sigma), and single colonies were identified by matrix-assisted laser desorption ionization–time of flight mass spectrometry (MALDI-TOF MS). Strain identity was confirmed from an axenic culture by internal transcribed spacer (ITS)/5.8 rDNA sequencing using primers ITS-1 and ITS-4 (8). The ITS sequence of strain YMX004033 was similar enough to be considered conspecific with the *K. humilis* strains with available sequences at the UNITE database (9); there were 13 different nucleotides (2.45%) compared with the type strain CBS5658 and 11 (2.07%) compared with a tequila fermentation strain also from Mexico [UWO(PS)92-219].

For short-read sequencing, genomic DNA was purified using the MasterPure DNA purification kit, and it was sequenced using the DNBSeq platform (BGI, China), generating 38,025,924 paired-end 150-bp reads. Adapters and low-quality reads (18.54%) were removed with fastp v0.20.0 (10). Additionally, high-molecular-weight DNA obtained with the Qiagen Genomic-tip (20/G) protocol, and fragmented to 20 kb with Covaris G-tubes, was used to prepare a sequencing library with the SQK-LSK109 Ligation protocol from Oxford Nanopore. Sequencing of this library was done in a FLO-MIN106D Spot-ON flow cell vR9. A total of 43,041 raw reads (457.38 Mb) with an estimated *N*_50_ value of 13.96 kb were obtained after base calling with Guppy v3.2.10. *De novo* genome assembly was performed with SMARTdenovo (11) after read trimming, filtering, and correction with Porechop v0.2, Filtlong v0.2, and Canu v1.8 (12), respectively. The final assembly was polished using two rounds of medaka v0.12.1 with the long reads, followed by three rounds of Pilon v1.23 (13) using the short reads, resulting in 21 scaffolds, with a total length of 14.96 Mb, an *N*_50_ value of 1.05 Mb, 49.16% GC content, and 30× mean coverage. Using BUSCO v4.0.5 (14), genome completeness was estimated to be 94.5% (Saccharomycetes odb10, *n* = 2,137) with 2% of duplicates. All programs were run with default parameters. The genome sequence described here will contribute to our understanding of the poorly described genome diversity of yeasts from Mesoamerica and will enable future research on this species of worldwide importance for food and beverage production.

### Data availability.

The data have been deposited at NCBI under BioProject number PRJNA765343, accession number JAIWYV000000000 (draft genome sequence), SRA numbers SRR16962313 (Nanopore reads) and SRR16016011 (DNBSeq reads), and accession number OK247582 (ITS sequence).
